# Osteoarthritis pathogenesis – a complex process that involves the entire joint

**Published:** 2014-03-25

**Authors:** GS Man, G Mologhianu

**Affiliations:** * “Carol Davila” University of Medicine and Pharmacy, Gastroenterology Department, Emergency University Hospital, Bucharest; ** “Carol Davila” University of Medicine and Pharmacy, Department of Rehabilitation Medicine, 3rd Rehabilitation Clinic of National Institute of Rehabilitation, Physical Medicine and Balneology, Bucharest

**Keywords:** osteoarthritis, cartilage, subchondral bone, synovium, menisci

## Abstract

Abstract

Osteoarthritis is the most common joint disorder and a major cause of disability with a major socio-economic impact. In these circumstances is very important to understand its pathogenesis. Although previous research focused primarily on changes in the articular cartilage, more recent studies have highlighted the importance of the subchondral bone, synovium, menisci, ligaments, periarticular muscles and nerves. Now osteoarthritis is viewed as a multifactorial disease affecting the whole joint.

Abbreviations: TNF-α – tumor necrosis factor alpha, IL-1 – interleukin -1, IL-6 – interleukin-6, COMP- cartilage oligomeric matrix protein, BSP - bone sialoprotein, MRI - magnetic resonance imaging, NTx - cross-linked N-telopeptide of type I collagen, CTx – C-telopeptide-cross-linked collagen type I, TGF-β – transforming growth factor beta, MMPs- matrix metaloproteinases, VEGF-vascular endothelial growth factor, bFGF - basic fibroblast growth factor.

Osteoarthritis is the most common chronic musculoskeletal disorder. Epidemiological studies estimate around 43 million affected patients in the United States alone and about 15% of the world population [**1**, **2**, **3**]. It is the leading cause of activity limitation and absenteeism among working-age adults and is associated with a significant decline in function among older individuals. In this conditions osteoarthritis is also a substantial economic burden, with annual medical care expenditures of billion dollars in the United States (**4**). Understanding of changes early in the development of osteoarthritis is important, since these changes could still be reversible, and therefore, preventive treatment could be initiated to slow or reverse further progression of the disease.

Initially, osteoarthritis has been considered to be a disease of articular cartilage, but recent research has indicated that the condition involves the entire joint [**5**, **6**, **7**]. The loss of articular cartilage has been thought to be the primary change, but a combination of cellular changes and biomechanical stresses causes several secondary changes, including subchondral bone remodeling, the formation of osteophytes, the development of bone marrow lesions, change in the synovium, joint capsule, ligaments and periarticular muscles, and meniscal tears and extrusion [**8**, **9**, **10**, **11**].

Articular cartilage

Normal adult articular cartilage is made up of extracellular matrix (water, collagen, proteoglycans and a very small component of calcium salt) and chondrocytes [**12**]. The turnover rate of collagen is relatively slow, whereas proteoglycan turnover is rapid [**13**]. The normal turnover of this matrix components is mediated by the chondrocytes, which synthetize these components and the proteolytic enzymes responsible for their breakdown. Chondrocytes are, in turn, influenced by a number of factors, including polypeptide growth factors and cytokines, structural and physical stimuli and even the components of the matrix itself [**14**].

Osteoarthritis result from failure of chondrocytes to maintain homeostasis between synthesis and degradation of these extracellular matrix components [**15**]. It is not known what initiates the imbalance between the degradation and the repair of cartilage. Trauma causing a microfracture or inflammation causing a slight increase in enzymatic activity may allow the formation of ”wear” particles, which could be then engulfed by resident macrophages [**16**]. At some point in time, the production of these ”wear” particles overwhelms the ability of the system to eliminate them and they become mediators of inflammation, stimulating the chondrocyte to release degradative enzymes. Molecules from breakdown of collagen and proteoglycan, also taken up by synovial macrophages, cause release of proinflammatory cytokines, like TNFα, IL-1 and IL-6. These cytokines can bind to chondrocyte receptors leading to further release of metalloproteinases and inhibition of type II collagen production, thus increasing cartilage degradation [**17**]. This disruption of homeostasis results in increased water content and decreased proteoglycan content of the extracellular matrix, weakening of the collagen network due to decreased synthesis of type II collagen and increased breakdown of pre-existing collagen [**18**]. Furthermore, there is increased apoptosis of chondrocytes.

Osteoarthritic cartilage is characterized by an increase in anabolic and catabolic activity. At first, compensatory mechanisms such as increased synthesis of matrix molecules (collagen, proteoglycans and hyaluronate) [**19**] and proliferation of chondrocytes in the deeper layers of the cartilage, are able to maintain the integrity of the articular cartilage, but in the end loss of chondrocytes and changes in extracellular matrix predominate and osteoarthritic changes develop.

Initial degenerative changes in the articular cartilage lead to cartilage softening, fibrillation zone of the superficial layers, fissuring and diminished cartilage thickness, but these changes become more pronounced with time, when articular cartilage thins to total destruction, eventually leaving the underlying subchondral bone plate completely exposed. All these changes in the articular cartilage are referred to as chondropathy.

Subchondral bone

It is not yet clear whether changes within subchondral bone precede changes in the articular cartilage or whether they occur in the disease progression, secondary to adaptation processes after changes in the biomechanical properties of the overlying articular cartilage. However, the two processes are closely related, as suggested by the concomitant increase in the levels of cartilage oligomeric matrix protein (COMP) and bone sialoprotein (BSP) in people with early osteoarthritis [**20**].

Subchondral bone consists of the subchondral bone plate and the underlying trabecular bone and bone marrow space. The subchondral bone plate consists of cortical bone and is separated from the articular cartilage by the zone of calcified cartilage.

Subchondral bone properties are modified through the cell mediated process of remodeling and modelling [**8**]. Bone remodeling includes the cupling of mechanisms that resorb bone and form new bone on a previously resorbed surface, whereas bone modeling is a mechanism that drives changes in the architecture and volume of bone via direct apposition to existing bone surfaces [**21**]. During the osteoarthritic process all of these mechanisms may be altered at some point in time resulting in subchondral bone structure changes.

 Changes in the bone include sclerotic changes and the development of bone marrow lesions that can be visualized by magnetic resonance imaging (MRI), and which seem to precede temporally and spatially, bone cysts in the subchondral compartment [**22**, **23**]. Thus, there is a progressive increase in the subchondral bone plate thickness, a modification in the architecture of subchondral trabecular bone, formation of new bone at the joint margins - osteophytes [**24**]. In subsets of patients with osteoarthritis, the indices of bone resorbtion showing loss of trabecular tissue indicated by the increase in cross-linked N-telopeptide of type I collagen (NTx) and C-telopeptide (CTx) [**25**], suggest a progressive loss of trabecular bone, not specifically of subchondral bone. In later stages, severe remodeling processes take place in particular in areas of advanced cartilage destruction, apart from extensive bone sclerosis (osteoid deposition), significant aseptic bone necrosis is a common feature of late-stage [20]. In areas of total cartilage destruction (the eburnated bone plate), synovial fluid gets access to the bone marrow and presumably leads to the bone cysts frequently seen in late stage disease [**20**]. Growth factors from the synovial fluid are probably involved in inducing fibrocytic and even chondrometaplastic changes, which lead to the ”cartilage nodules” characteristic for late-stage disease [**20**].

In osteoarthritic subchondral bone, type I of collagen is elevated, but this collagen content is abnormal and this leads to abnormal mineralization. In normal bone, type I collagen is composed of a heterotrimer of α1 and α2 chains at an average ratio of 2.4:1. In osteoarthritic bone tissue this ratio varied between 4:1 and 17:1 (**26**), and this appears to be responsible for the abnormal mineralization pattern. Elevated TGF β1 levels in osteoarthritic osteoblast are responsible, in part, for the abnormal ratio of collagen I α1 to collagen I α2 and for the abnormal production of mature type I collagen [**27**]. Thus osteoarthritic subchondral bone has an increased osteoid collagen matrix and an abnormal mineralization resulting in a hypomineralization of this tissue. Although the subchondral bone tissue is hypomineralized in osteoarthritis, the increase in trabecular number and volume compensates for this situation, thus providing an apparent stiffer structure [6]. With alteration in its properties, subchondral bone may be less able to absorb and dissipate energy, thereby increasing forces transmitted through the joint and predisposing the articular surface to deformation [**28**]. Subchondral bone attrition may be caused by altered mechanical loading resulting in subchondral remodeling and is associated with concomitant bone marrow lessions [**29**]. Bone attrition is evaluated at conventional radiography as loss of bone density [**30**] or, at MRI, as flattening/depression of the articular cortex [**31**]. MRI studies have demonstrated that these bone lesions themselves are associated with development and worsening of cartilage loss [**32**].

Bone marrow lesions are degenerative lesions consisting of edema, bone marrow necrosis, fibrosis and trabecular abnormalities [**33**, **34**]. They are a marker for increased metabolic activity and their persistence is associated with local cartilage damage [**35**, **36**]. Bone marrow lesions presence, incidence and progression have been associated with development and worsening of cartilage loss, including in locations adiacent to the bone marrow lesions [**28**, **37**, **38**].

The exact origin of subchondral cyst-like lesion remains to be elucidated, but it is currently thought that they result from synovial fluid intrusion as a consequence of elevated intra-articular pressure [**33**]. They may be present within or adjacent to a bone marrow lesions [**39**].

Synovial membrane

It remains unclear whether the morphological changes that occur in the osteoarthritic synovial membrane are primary or whether they are the result of joint inflammation, cartilage degradation and lesions of the subchondral bone [**40**]. Histologically, the synovial membrane of osteoarthritic joints commonly exhibits hyperplasia of the lining cell layer occasionally accompanied by focal infiltration of lymphocytes and monocytes in sublining layers [**41**]. Synovitis is belived to be induced at first by the cartilage matrix proteolytic degradation products that produce wear particles and soluble cartilage-specific neo-antigens, as well as other factors including microcrystals and abnormal mechanical stress [**6**]. These components are released into the synovial fluid and are phagocyted by synovial lining macrophages, perpetuating the inflammation of the synovial membrane through the synthesis of mediators, which in turn diffuse through the synovial fluid into the cartilage, and create a vicious circle, with increased cartilage degradation, and subsequently produce more inflammation [**6**]. This also explains the increase in the amount of CD68-positive type A synoviocytes (macrophage-like), which have phagocytic capacity, in the synovial lining layer [**20**]. Patients with osteoarthritis experience thickening of the synovial lining cell layer, increased vascularity and inflammatory cell infiltration of the synovial membranes, with the most marked changes occurring in advanced osteoarthritis. Studies of the changes in the synovium that occur at various stages of osteoarthritis have found that the amount of fibrin deposited in the synovial membrane and the degree of leukocyte infiltration are correlated with disease severity [**42**].

Synovial cells and osteoarthritic chondrocytes both produces large quantities of matrix metaloproteinases (MMPs), like MMP-1, MMP-3, MMP-9 and MMP-13 [**43**]. Synoviocytes are able to secrete not only proteolytic enzymes, but also proinflammatory cytokines (IL-1β, IL-6, TNF-α), which are thought to mediate the progression and pain associated with this disease [**44**]. Adipokines, such as resistin [**45**] are also expressed by the synovium during osteoarthritis. Thus, synovial tissue seems to be the main source of adipokines in the osteoarthritic joint [**46**]. Osteopontin, a cytokine whose increased expression levels have been correlated with disease severity, is expressed in high quantity by synovial tissue in osteoarthritis [**47**]. The synovium produces some of the chemokines and metalloproteinases that degrade cartilage, even though the cartilage itself produces most of these destructive molecules in a vicious autocrine and paracrine fashion [**48**]. In turn, cartilage breakdown products, resulting from mechanical or enzymatic destruction, can provoke the release of collagenase and other hydrolytic enzymes from synovial cells and lead to vascular hyperplasia in osteoarthritic synovial membranes [**49**].

Synovial neovascularization may be largely driven by synovitis as inflammatory cells such as macrophages can themselves secrete pro-angiogenic factors, but also secrete factors that stimulate other cells, such as endothelial cells and fibroblasts, to produce vascular endothelial growth factor (VEGF), basic fibroblast growth factor (bFGF) and other factors that further promote angiogenesis [**49**, **50**]. Then, the blood vessel permeability and up-regulation of adhesion molecules that are seen as part of angiogenesis perpetuates the inflammatory response [**47**]. Angiogenesis in the synovium is closely associated with chronic synovitis and may occur at all stages of osteoarthritis [**51**].

Menisci

Meniscal degeneration is commonly seen in osteoarthritis, where menisci appear torn, fissured, fragmented, macerated or completely destroyed [**52**]. Degeneration of menisci initiates within the substance of the tissue rather than surface. Tissue fibrillation and disruption is first seen at the inner rim, which spreads to the articular surfaces of the meniscus over time, and progresses to total disruption or loss of meniscus tissue mainly in the avascular zone [**53**]. Type I collagen content decrease gradually from the surface zone to the middle and the deep zone of osteoarthritic meniscus [**54**]. Different from type I collagen, the decrease of type II collagen content is severe in the surface zone, and also prominent in the middle and deep zones of osteoarthritic meniscus [**54**]. In turn, proteoglycan content increase in osteoarthritic menisci when compare to normal menisci [**54**]. All these intrameniscal changes correlated with peri-meniscal synovitis, calcification not limited to the outer, peripheral portion of the menisci [**55**, **56**] contribute to meniscal degeneration and reduced meniscal tensile strength. The meniscus is less able to withstand loading and force transmission during normal movements of the joint, further leading to degenerative tears [**57**]. Meniscal tears are often accompanied by varying degrees of meniscal extrusion [**58**]. The tear might be a preceding feature of incipient osteoarthritis, and meniscus damage and extrusion often have a key role in the structural progression of the disease [**59**].

In conclusion, osteoarthritis is a multifactorial disease of whole joint, with a complex pathomechanism involving interaction between the multiple joint tissue. Knowing of this complex process of producing osteoarthritis is essential for development of new methods of diagnostic and treatment.

Disclosures: authores declare no disclosure.
